# What Are the Preferences in Patient-Physician Communication Among Total Joint Arthroplasty Patients?

**DOI:** 10.1016/j.artd.2025.101682

**Published:** 2025-04-21

**Authors:** Siddhartha Dandamudi, Kyleen Jan, Ilyass Majji, Anne DeBenedetti, Omar Behery, Brett R. Levine

**Affiliations:** a Department of Orthopaedics, Rush University Medical Center, Chicago, IL, USA; b Georgetown University School of Medicine, MedStar Georgetown University Hospital, Washington, D.C, USA

**Keywords:** Arthroplasty, Satisfaction, Communication

## Abstract

**Background:**

Effective communication is essential in fostering a strong physician-patient relationship. With a growing number of patient engagement platforms, understanding communication preferences is crucial in guiding patient contact strategies more effectively. The goal of this study is to explore communication medium preferences of hip and knee arthroplasty patients.

**Methods:**

A 10-question survey focused on patient communication preferences with the surgical team was distributed anonymously to patients at clinic visits of multiple arthroplasty surgeons at a large academic center. No identifying information was collected.

**Results:**

Four hundred seventy-two responses were collected. Of the patients, 95.6% were willing to share their phone number with the surgical team. Of those responding, 53.5% and 35.4% indicated that a phone call and text, respectively, were their preferred communication medium. A majority (93%) of respondents had positive feelings towards receiving 1-way message updates with reminders, videos, and expected milestones perioperatively. Most patients (92.5%) want an open line of communication with the surgical team, and almost every respondent (99.6%) believes that the surgeon or insurance company is responsible financially for this means of communication or patient engagement platform.

**Conclusions:**

Our data suggest that direct communication via phone calls and texts are the preferred media of communication with arthroplasty patients. Financially, surgeons should be aware that engagement platforms may add to practice costs. However, incorporating these insights into practice can enhance patient satisfaction, ultimately leading to better outcomes and more efficient care. Understanding these preferences may afford clearer, more effective interactions with patients and potentially improve overall engagement and outcome data collection.

## Introduction

Patient satisfaction is a major outcome goal in delivering clinical orthopaedic care including the hip and knee arthroplasty population. Effective provider-patient communication is a key component in fostering a meaningful connection between surgeons and their patients [1]. As the landscape of health care continues to evolve with technological advancements and increasing utilization of various patient engagement platforms, understanding and adequately addressing patient communication preferences have become increasingly pivotal in delivering personalized and patient-centered care [2]. Hip and knee arthroplasty patients, who often require longitudinal care and treatment for musculoskeletal conditions, rely heavily on effective communication with their health-care providers to navigate their treatment journey successfully [3]. Provider trust and direct patient communication can significantly impact patient outcomes, protocol adherence, and overall satisfaction with the care received [1]. Additionally, an open line of communication may provide better access to completing patient-reported outcome forms.

Understanding patient communication preferences is critical for enhancing satisfaction with care, yet this area remains underexplored. Despite the growing recognition of the impact communication has on patient experiences, there is a significant gap in our understanding of how specific preferences—such as preferred channels and frequencies of communication—affect overall satisfaction and engagement [4]. A comprehensive investigation into these preferences is essential. By elucidating how patients wish to be communicated with, we can better align health-care practices and patient needs, ultimately leading to more personalized, effective care and improved patient satisfaction.

This study aims to describe the communication medium preferences of hip and knee arthroplasty patients, seeking to identify the most preferred methods through which patients wish to interact with their health-care providers. By gaining a deeper understanding of how patients prefer to receive and share information relevant to their care from their provider team, practice patterns may be tailored to improve communication methods to address the needs and preferences of these patients.

## Material and methods

This cross-sectional survey study was approved by the institutional review board. A 10-question survey was created by the senior authors with key questions developed to gather communication preferences among hip and knee arthroplasty patients (Table 1, Table 2). Inclusion criteria in this study included all new and established patients who agreed to take part in the study and filled out the survey. It was not possible to determine whether new or postsurgical (established) patients completed each survey. All patients were given the choice to opt out prior to filling out the survey. Any patient who refused to fill out the survey was excluded from this study.

**Table 1:** Patient answered questions regarding communication with providers.

Question	Yes %	No %
1. Are you concerned about sharing data with your doctor?	5.5	94.5
2. Are you willing to share this data (cell phone number, email address) with your surgeon and their team?	95.6	4.4
3. Are you okay filling out surveys on your progress via the internet/email?	81.7	18.3
4. Do you think an open line of telehealth and e-communication should be available with your surgical team?	92.5	7.5
5. Is this a form of communication you would envision yourself using?	82	18
6. Do you think the surgical team should be compensated for having an open line for telehealth and e-communications?	70.5	29.5
7. How do you think this open line of communication should be paid for?^a^	-	-
8. How do you feel about a service that sends you 1-way messages with updates, reminders, videos, and milestones before and after surgery from your surgeon and their team?^a^	-	-
9. How do you feel about participation in medical research?^a^	-	-

a The answers to the following questions were not “yes” or “no.” The responses are described in the text.

**Table 2:** Answers to “As a patient, what ways are preferred for communication with your surgical team?”

Medium	Most preferred (%)	Least preferred (%)
Email	26.7	17.4
Text	35.4	5.3
Phone call	53.5	12.8
Patient portal	9.4	55.8

Rank 1 = most preferred, 2 = second preferred, 3 = third preferred, 4 = least preferred.

The 10-question survey seeks to understand patients' attitudes towards various aspects of communication and data sharing in the context of surgical care. It explores concerns about sharing personal data, such as cell phone numbers and email addresses, and assesses the willingness of patients to complete progress surveys online and utilize telehealth services for ongoing communication with their surgical team. Other questions in the survey inquire about the potential benefits and acceptability of receiving updates, reminders, and educational content from the surgical team through 1-way messaging. Additionally, it gauges opinions on whether surgical teams should be compensated for providing telehealth services and how such services should be funded. Finally, it touches on patients' openness to participating in medical research.

The survey used in this study explored patients' concerns about sharing personal data with their physician and their willingness to do so through online progress surveys or email. It also assessed whether patients believe that all patients should have access to continuous e-communication with their surgical team and their preferred communication methods. Additionally, the survey inquired about whether surgical teams should be compensated for maintaining this open line of communication and how such compensation should be structured. Furthermore, it gathered information on patients' willingness to participate in medical research. It is important to note that the standard practice of our institution is to offer services of 1-way updates, reminders, and videos before and after surgery, but patients are not obligated to use this service. Rejecting the opportunity to participate in e-communication does not prohibit a patient from having surgery if all other clinical parameters and requirements are met.

Over a 3-month period, surveys were distributed to patients of multiple fellowship-trained hip and knee arthroplasty surgeons at a single, high-volume, tertiary care academic center in a clinic setting. Patient participation was voluntary, where all patients, new and established, were handed the survey and given the opportunity to refuse participation. These were all anonymous, and patients were instructed not to leave any patient-identifying information. Surveys were distributed to patients in the clinic prior to their regularly scheduled appointment along with intake paperwork. Front desk staff collected surveys alongside intake paperwork so no answers were changed by speaking to the provider or medical staff.

Surveys were collected, and data were extracted by junior authors into Microsoft Excel (Version 16.85, Microsoft, Redmond, WA) for analysis. Descriptive statistics summarizing patient responses to the survey were calculated, and qualitative data are presented as percentages of responses.

## Results

Four hundred seventy-two patients completed the survey. Responses to the survey questions are shown in Table 1. The survey results reveal that a minimal 5.5% of patients are concerned about sharing their data with their doctor. Conversely, a substantial 94.5% of respondents have no such concerns. When it comes to sharing contact information, an overwhelming 95.6% of patients are willing to provide their cell phone numbers and email addresses to their surgeon and their team. Furthermore, 81.7% agree with completing progress surveys online or via email, and a significant 92.5% of patients advocate for the availability of telehealth and e-communication channels with their surgical team, and 82% see themselves actively using these forms of communication. Regarding financial considerations, 70.5% of patients support the idea that surgical teams should receive compensation for maintaining an open line of telehealth and e-communication, while 29.5% oppose this notion.

In response to the survey, 47.5% of respondents believed that surgeons should bear the cost of maintaining open lines of communication, while 52.1% felt that this should be covered by insurance, and only 0.42% believed the expense should be the patient's responsibility. Among the 472 surveyed patients, 41.2% expressed strong satisfaction with a service that provides 1-way updates, reminders, and videos before and after surgery, and 51.8% indicated general approval. In contrast, 5.9% were dissatisfied with the service, and 1.2% strongly disliked it. The survey also assessed patient willingness to engage in medical research; 44.8% were open to participation, 36.5% were favorable but uncertain, 14.3% were unwilling, and 4.4% were strongly opposed to participating in any form of medical research.

In the last question of the survey, patients were asked to rank their preferences on mediums of communication. The results displayed in Table 2 indicate that phone calls are the most preferred method of communication with surgical teams, favored by 53.5% of patients, while only 12.8% find them least preferred. Text messages are also popular, with 35.4% ranking them as their top choice and just 5.3% considering them least preferred. Email is the third most preferred method, chosen by 26.7% as their first preference but with 17.4% listing it as least preferred. The patient portal is the least favored option, with only 9.4% of patients ranking it most preferred and 55.8% marking it as least preferred. These insights highlight the importance of prioritizing phone calls and texts in communication strategies to better align with patient preferences and enhance satisfaction.

## Discussion

Integrating telehealth and electronic provider-patient communication into surgical care is increasingly recognized as a valuable tool for enhancing patient outcomes and satisfaction. Understanding patient perspectives on data sharing, communication preferences, and compensation for telehealth services is essential for implementing effective practices. This study descriptively highlights several key insights into patient perspectives toward telehealth and electronic communication in orthopaedic surgical care. The high level of trust in data sharing with doctors, coupled with a strong willingness to provide contact information, underscores the importance of secure and transparent communication channels. Most patients in this study were found to be comfortable with digital follow-up methods and support the availability of telehealth services, suggesting patient acceptance of the use of technology in health-care communication.

With 94.5% of patients not reporting concerns about sharing data with their provider, this suggests patient trust in health-care providers in handling their personal information. This trust may be a requisite aspect in the successful implementation of telehealth communication services, as patients need to feel confident that their sensitive information will be protected [5]. Similarly, 95.6% of patients indicated their willingness to share contact information such as cell phone numbers and email addresses with their surgical teams, demonstrating a strong patient desire for direct and potentially more efficient communication with health-care providers. This trend towards digital communication and patient engagement can be leveraged to streamline processes and ensure timely updates and interventions, which are crucial in surgical care [6]. Furthermore, as patient-reported outcome measures (PROMs) collection and reporting requirements evolve, these digital communication mediums can be increasingly utilized to enhance data collection and compliance with the constantly changing mandates from the Centers for Medicare & Medicaid Services [7]. This adaptability will be the key in maintaining effective and efficient practices in the face of regulatory changes.

The results have also revealed that patients recognize the need for compensation for telehealth services, preferring these costs to be absorbed by insurance or included in the surgical fee rather than as an additional expense. This preference aligns with efforts to integrate telehealth into standard care practices without placing a financial burden on patients. The acceptance of internet/email-based surveys by 81.7% of patients indicates a broad readiness to engage with digital follow-up methods, which is particularly significant for enhancing postoperative care through continuous monitoring and feedback [6]. However, 18.3% of patients were uncomfortable with this mode of communication. This could be due to several factors that we have not investigated in this study, such as age of the patient, technology literacy, and preferred language of communication [2]. This strengthens the need for alternative follow-up options in clinical practice to ensure inclusivity. Developing user-friendly, language-appropriate, and secure platforms for digital surveys can further increase acceptance and participation, thereby enhancing the overall quality of care [8].

Furthermore, there was significant support (92.5%) for the availability of telehealth and e-communication channels, underscoring their importance in modern health care. This could be attributed to patients recognizing the benefits of direct and immediate access to their surgical teams, particularly for addressing postoperative concerns and reducing the need for in-person visits [9]. Implementing robust telehealth systems can improve patient satisfaction, reduce hospital readmissions, and optimize resource utilization within health-care facilities [3]. However, as the costs associated with running clinical practices continue to increase and the number of patients requiring total joint reconstruction procedures continues to rise, this could ultimately result in a considerable financial strain on orthopaedic clinical practices [10]. To navigate these challenges, exploring strategies for cost management and efficiency improvements may prove essential to continue delivering high-quality care while remaining financially sustainable.

Limitations of this study include that the data were collected from a single urban academic center, limiting its external validity for different regions or health-care settings where digital communication is not feasible. Second, the study did not collect demographic data and thus cannot evaluate whether there are any patient-related factors that influence responses regarding communication medium preferences. Also, the voluntary survey-based study design makes the results prone to nonresponse and convenience bias associated with this design.

## Conclusions

This study provides valuable insights into patient perspectives on telehealth and e-communication in surgical care. The positive attitudes towards data sharing, willingness to engage in digital communication, and support for telehealth services underscore the readiness for integrating these technologies into routine practice. By addressing patient preferences and concerns, health-care providers may potentially enhance the effectiveness and acceptance of telehealth services, ultimately improving patient outcomes and satisfaction. Future research, including comparative studies, should explore the long-term impacts of telehealth on patient outcomes and cost-effectiveness to further inform health-care practices and policies.

## Conflicts of interest

A. DeBenedetti is a paid consultant for JointMedica Limited. B.R. Levine receives IP royalties from Link Orthopaedics; is a paid consultant for Enovis, Link Orthopaedics, Merete, and Zimmer Biomet; receives research support from Zimmer Biomet; is an editorial or governing board member of Arthroplasty Today, Elsevier, Human Kinetics, SLACK Incorporated, Wolters Kluwer Health, and Lippincott Williams & Wilkins; and is a board or committee member of AAOS, the American Association of Hip and Knee Surgeons, the Knee Society, and MAOA. All other authors declare no potential conflicts of interest.

For full disclosure statements refer to https://doi.org/10.1016/j.artd.2025.101682.

## CRediT authorship contribution statement

**Siddhartha Dandamudi:** Writing – review & editing, Writing – original draft, Supervision, Resources, Project administration, Methodology, Investigation, Formal analysis, Data curation, Conceptualization. **Kyleen Jan:** Writing – review & editing, Writing – original draft, Software, Methodology, Investigation, Formal analysis, Data curation, Conceptualization. **Ilyass Majji:** Writing – review & editing, Writing – original draft, Visualization, Methodology, Formal analysis, Data curation, Conceptualization. **Anne DeBenedetti:** Writing – review & editing, Supervision, Project administration, Methodology, Data curation, Conceptualization. **Omar Behery:** Writing – review & editing, Writing – original draft, Visualization, Validation, Supervision, Project administration, Methodology, Investigation, Formal analysis, Conceptualization. **Brett R. Levine:** Writing – review & editing, Writing – original draft, Visualization, Validation, Supervision, Project administration, Methodology, Investigation, Formal analysis, Conceptualization.

## Appendix A Supplementary data

Conflict of Interest Statement for DeBenedetti

Conflict of Interest Statement for Majji

Conflict of Interest Statement for Behery

Conflict of Interest Statement for Levine

Conflict of Interest Statement for Jan

Conflict of Interest Statement for Dandamudi
