# Supplementary material for: What Are the Preferences in Patient-Physician Communication Among Total Joint Arthroplasty Patients?
Source: Arthroplast Today. 2025 Apr 21;33:101682. doi: 10.1016/j.artd.2025.101682 (PMC12051045; doi:10.1016/j.artd.2025.101682)

# **INDIVIDUAL CONFLICT OF INTEREST STATEMENT**

***American Association of Hip and Knee Surgeons***

(Adopted from the American Academy of Orthopaedic Surgeons disclosure statement)

The following form **must be filled out completely and submitted by each author (example, 6 authors, 6 forms).**

**All items require a response. If there is no relevant disclosure for a given item, enter "*None*.”**

What Are the Preferences in Patient-Physician Communication Among Total Joint Arthroplasty Patients

**Manuscript Title**

1. Royalties from a company or supplier (The following conflicts were disclosed)

**None**

2. Speakers bureau/paid presentations for a company or supplier (The following conflicts were disclosed)

**None**

3A. Paid employee for a company or supplier (The following conflicts were disclosed)

**None**

3B. Paid consultant for a company or supplier (The following conflicts were disclosed)

**JointMedica Limited**

3C. Unpaid consultants for a company or supplier (The following conflicts were disclosed)

**None**

4. Stock or stock options in a company or supplier (The following conflicts were disclosed)

**None**

5. Research support from a company or supplier as a Principal Investigator (The following conflicts were disclosed)

**None**

6. Other financial or material support from a company or supplier (The following conflicts were disclosed)

**None**

7. Royalties, financial or material support from publishers (The following conflicts were disclosed)

**None**

8. Medical/Orthopaedic publications editorial/governing board (The following conflicts were disclosed)

**None**

9. Board member/committee appointments for a society (The following conflicts were disclosed)

**None**

**Each author must sign AND print or type his/her name, date and submit a separate form**

In addition, one BLINDED Conflict of Interest form (no author names used) should be submitted per manuscript with all author disclosures.

Anne DeBenedetti, MSc 9/19/2024


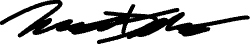


Author Name (Print or Type) Author Signature Date


Supplement: Conflict of Interest Statement for DeBenedetti [file mmc1.docx]
