# Supplementary material for: What Are the Preferences in Patient-Physician Communication Among Total Joint Arthroplasty Patients?
Source: Arthroplast Today. 2025 Apr 21;33:101682. doi: 10.1016/j.artd.2025.101682 (PMC12051045; doi:10.1016/j.artd.2025.101682)
Supplement: Conflict of Interest Statement for Behery [file mmc3.docx]

# CONFLICT OF INTEREST STATEMENT

***American Association of Hip and Knee Surgeons***

(Adopted from the American Academy of Orthopaedic Surgeons disclosure statement)

**What Are the Preferences in Patient-Physician Communication Among Total Joint Arthroplasty Patients**

Manuscript Title

1. Royalties from a company or supplier (The following conflicts were disclosed)

*None*

2. Speakers bureau/paid presentations for a company or supplier (The following conflicts were disclosed)

*None*

3A. Paid employee for a company or supplier (The following conflicts were disclosed)

*None*

3B. Paid consultant for a company or supplier (The following conflicts were disclosed)

*None*

3C. Unpaid consultants for a company or supplier (The following conflicts were disclosed)

*None*

4. Stock or stock options in a company or supplier (The following conflicts were disclosed)

*None*

5. Research support from a company or supplier as a Principal Investigator (The following conflicts were disclosed)

*None*

6. Other financial or material support from a company or supplier (The following conflicts were disclosed)

*None*

7. Royalties, financial or material support from publishers (The following conflicts were disclosed)

*None*

8. Medical/Orthopaedic publications editorial/governing board (The following conflicts were disclosed)

*None*

9. Board member/committee appointments for a society (The following conflicts were disclosed)

*None*

**Each author must sign AND print or type his/her name, date and submit a separate form**

In addition, one BLINDED Conflict of Interest form (no author names used) should be submitted per manuscript with all author disclosures.

Omar Behery
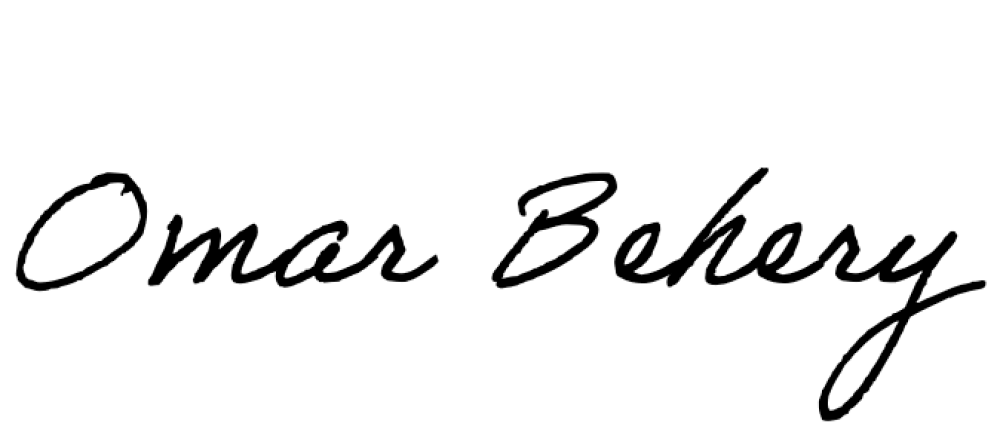
 9/14/2024

Author Name (Print or Type) Author Signature Date
